# The Desaturase Gene Family is Crucially Required for Fatty Acid Metabolism and Survival of the Brown Planthopper, *Nilaparvata lugens*

**DOI:** 10.3390/ijms20061369

**Published:** 2019-03-19

**Authors:** Jia-mei Zeng, Wen-feng Ye, Ali Noman, Ricardo A.R. Machado, Yong-gen Lou

**Affiliations:** 1 State Key Laboratory of Rice Biology, Institute of Insect Science, Zhejiang University, Hangzhou 310058, China; tsengchiamei@163.com (J.-m.Z.); wenfeng.ye@unine.ch (W.-f.Y.); alinoman@gcuf.edu.pk (A.N.); 2 Laboratory of Fundamental and Applied Research in Chemical Ecology, University of Neuchâtel, Neuchâtel 2000, Switzerland; 3 Department of Botany, Government College University, Faisalabad 38040, Pakistan; 4 Institute of Plant Sciences, University of Bern, Bern 3013, Switzerland; ricardo.machado@ips.unibe.ch

**Keywords:** Desaturase, Fatty acid metabolism, Function, Gene family, *Nilaparvata lugens*, Rice

## Abstract

Desaturases are essentially required for unsaturated fatty acid (UFA) biosynthesis. We identified 10 genes encoding putative desaturases in the transcriptome database of the brown planthopper (BPH), *Nilaparvata lugens*. These include eight First Desaturase family genes, one cytochrome b5 fused desaturase gene (*Nlug-Cytb5r*) and one Sphingolipid Desaturase gene (*Nlug-ifc*). Transcript level profiling revealed significant variation in the expression patterns of these genes across tissues and developmental stages, which occur in a gene-specific manner. Interestingly, their expression was also modulated by the insect food source: the mRNA levels of *Nlug-desatC* and *Nlug-Cytb5r* were down-regulated, but the expression level of *Nlug-desatA1-b* and *Nlug-desatA1-c* were elevated in the BPH fed on the resistant rice variety Babawee as compared to the non-resistant variety Taichun Native 1 (TN1). Silencing *Nlug-desatA1-b*, *Nlug-desatA1-c*, or *Nlug-Ifc* reduced fatty acid composition and abundance in female BPH 1-d-old-adults compared to controls. Whereas, single knockdown of all ten desaturase genes significantly increased mortality of BPH nymphs compared with controls. Of the ten desaturase genes, knockdown of *Nlug-desatA1-b* and *Nlug-desatA2* caused the highest mortality in BPH (91% and 97%, respectively). Our findings offer a base for expression and functional characterization of newly identified desaturase genes in BPH, and may contribute to RNA interference-based pest management strategies.

## 1. Introduction

In eukaryotes, unsaturated fatty acids (UFA) perform diverse biological functions such as regulation of membrane fluidity [1,2], energy storage [3,4] and signaling [5,6]. In biosynthesis of UFAs, desaturases are the key enzymes. These catalyze the induction of unsaturated bonds into an acyl chain at specific positions. Desaturases are categorized into two independent phylogenetic groups namely soluble acyl-acyl carrier protein (ACP) desaturases and membrane-bound fatty acid desaturases [7]. ACP desaturases are mainly found in the plastids of higher plants. They specifically catalyze the conversion of saturated fatty acids into monounsaturated acids, e.g., oleic acid synthesis (conversion of C18:0 to C18:1) [8]. Membrane-bound fatty acid desaturases are the primary group of proteins responsible for desaturation in eukaryotes and bacteria. Typical membrane-bound fatty acid desaturases have three conserved histidine (His) box regions i.e., HX_(3-4)_H, HX_(2-3)_HH and H/QX_(2-3)_HH (H, histidine; X, unknown amino acid residues; Q, serine; subscript means the number of amino acid residues) [7,9]. According to substrate type (glycerolipid-linked or coenzyme A (CoA)-linked fatty acid substrates), membrane-bound fatty acid desaturases can be further classified into membrane-bound acyl-lipid desaturases and membrane-bound acyl-CoA desaturases. The membrane-bound acyl-lipid desaturases have been predominantly reported in plants, cyanobacteria [10] and *Caenorhabditis elegans* [11]. Furthermore, acyl-CoA desaturases are ubiquitous among animals, fungi and many bacteria [7]. Based on phylogenetic and motif analyses, the membrane-bound fatty acid desaturases can also be divided into four functionally distinct subfamilies. First Desaturases are responsible for introducing the first double bond into the saturated acyl chain mainly at the Δ9 or Δ11 position. Omega Desaturases introduce a double bond between an existing double bond and the acyl end at the Δ12 or Δ15 position. Front-end Desaturases introduce a double bond between an existing double bond and the carboxyl end inclusive of Δ4, Δ5, Δ6 and bifunctional Δ6/sphingolipid Δ8 desaturases. Sphingolipid Desaturases introduce a double bond into sphingolipids at the Δ4 position [12].

Insect desaturases belong to the acyl-CoA desaturase category. Helmkampf et al. [13] analyzed the acyl-CoA desaturase gene families of 15 insect species. They reported, on the basis of phylogenetic analyses, that insect desaturases characteristically constitute eight groups (Desat A1, A2, B, C, D, E, Ifc (*infertile crescent*) and Cyt-b5-r (*Cytochrome b5-related*)). Except for Ifc and Cyt-b5-r, all groups belong to the subfamily First Desaturases with Δ9 or Δ11 activity [12] while the Ifc group is a member of the Sphingolipid Desaturases with Δ4 activity [14]. The phylogenetic relationship of Cyt-b5-r to other desaturase genes is not well understood; however, previous studies revealed that the Cyt-b5-r group is probably involved in immune defense activation [15] and cold adaptation [16,17] in insects, and the Ifc protein plays a role in the reproduction of *Drosophila* [18].

Insect acyl-CoA desaturases are of immense significance in biosynthesis and perception of semiochemicals [19], cold tolerance [20], defensive fatty acids biosynthesis [21], feeding behavior [22] and larval development [23]. Similar to stearoyl-CoA desaturase SCD1 in mice [24] and Δ9 desaturases in *C. elegans* [25], the *desat1* gene in *Drosophila melanogaster* is important for regulating total fatty acid levels, as well as larval molt and development [22,23,26]. The functions of insect acyl-CoA desaturases have been much studied because of their crucial role in the biosynthesis of pheromones in many Lepidopteran species [27,28], *D. melanogaster* [29,30] and some bumblebee species [31]. To date, desaturases are well-studied in several Lepidopteran, Dipteran, and Hymenopteran species, and several desaturases have been identified and characterized across other insect orders as well. For instance, desaturases Δ5, Δ9 and Δ12 in the flour beetle *Tribilium castaneum* (Coleoptera) [32,33] and Δ9 desaturase in the house cricket *Acheta domesticus* (Orthoptera) [34] have been cloned and functionally characterized. In contrast, we face a shortage of information about the desaturases of Hemipteran insects, although recently a metathoracic scent gland desaturase has been found that is involved in female sexual attractiveness in the plant bug *Adelphocoris suturalis* [35].

The rice brown planthopper (BPH, *Nilaparvata lugens* Stål) (Hemiptera) is one of the most devastating pests of the rice plant (*Oryza sativa* L.) [36]. Besides its role as a vector of plant pathogens, e.g., rice ragged stunt virus (RRSV) and rice grassy stunt virus (RGSV) [37,38], BPH extract nutrition from rice plants, which leads to yellowing and drying of the plant [39,40]. In Asia, BPH causes substantial losses in rice yields annually. In 2005 and 2008, a yield loss of 2.7 million tons of rice in China was due to BPH infestation, while a yield loss of 2.7 million tons of rice in Vietnam was mainly due to RRSV and RGSV transmitted by BPH [36]. Due to the crucial role of desaturases in insects, these genes could be targeted within RNA interference-based pest management strategies. Many desaturases have been well studied in Lepidopteran and Dipteran species, but, unfortunately, very few desaturases have been identified in Hemipteran species. Moreover, how these enzymes affect the growth, development and reproduction of insects still needs extensive investigation. Therefore, we identified and annotated the desaturase family genes in the BPH genome. By combining sequence alignments, phylogenetic construction, expression pattern analysis and RNA interference (RNAi), some functions of BPH desaturase genes have been elucidated. We report that desaturase genes play critical role in the survival and fatty acid metabolism of BPH. These findings improve our understanding of the molecular mechanisms underlying UFA metabolism and survival in BPH.

## 2. Results

### 2.1. Brown Planthopper Genome Contains 10 Putative acyl-CoA Desaturases

**Figure 1:**
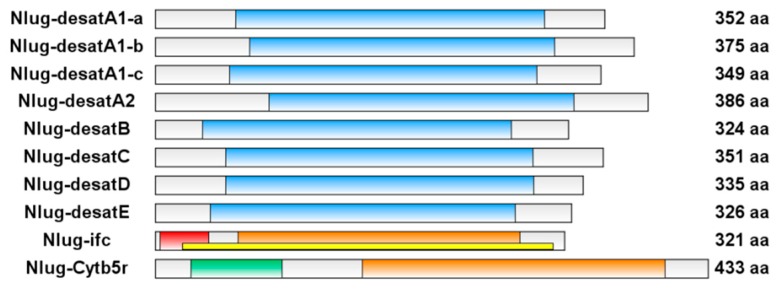
Schematic diagram of the domain architecture of 10 *Nilaparvata lugens* desaturase (Nlug-desat) genes. The deduced amino acid (aa) sequences were used to predict the domain architecture of Nlug-desats with Batch CD-search (National Center for Biotechnology Information) and InterProScan. The blue boxes indicate the Δ9-fatty acid desaturase-like domain (Δ9-FADS-like, cd03505). The red and the yellow box indicates the sphingolipid Δ4-desaturase, N-terminal (IPR013866) and Δ4-sphingolipid fatty acid desaturase-like domain (Δ4-sphingolipid-FADS-like, cd03508), respectively. The green and the orange box indicate the cytochrome b5-like heme/steroid binding domain (Cyt-b5, pfam00173) and the fatty acid desaturase domain (IPR005804), respectively.

Using all ten acyl-CoA desaturase genes from *D. melanogaster* as queries, BLAST searches of the BPH fat body transcriptomic databases [41] identified 10 putative desaturase genes. All the 10 genes were further confirmed by RT-PCR and sequencing, and uploaded to GenBank (accession numbers MH271225-MH271234). Eight putative BPH desaturases (Nlug-desatA1-a, Nlug-desatA1-b, Nlug-desatA1-c, Nlug-desatA2, Nlug-desatB, Nlug-desatC, and Nlug-desatD, and Nlug-desatE) possess a Δ9-fatty acid desaturase-like conserved domain (Δ9-FADS-like, cd03505) and belong to the First Desaturase subfamily. In contrast, the Nlug-ifc gene contains a sphingolipid Δ4-desaturase N-terminal domain (IPR013866) in the 5′ region, and a fatty acid desaturase domain (IPR005804) in the 3′ region of the amino acid (aa) sequence, and a Δ4-sphingolipid fatty acid desaturase-like domain (Δ4-sphingolipid-FADS-like, cd03508) across the aa sequence. Furthermore, the Nlug-Cytb5r gene contains a cytochrome b5-like heme/steroid binding domain (Cyt-b5, pfam00173) and a fatty acid desaturase domain (IPR005804) in the 5′ and 3′ region of the aa sequence, respectively (Figure 1).

**Figure 2:**
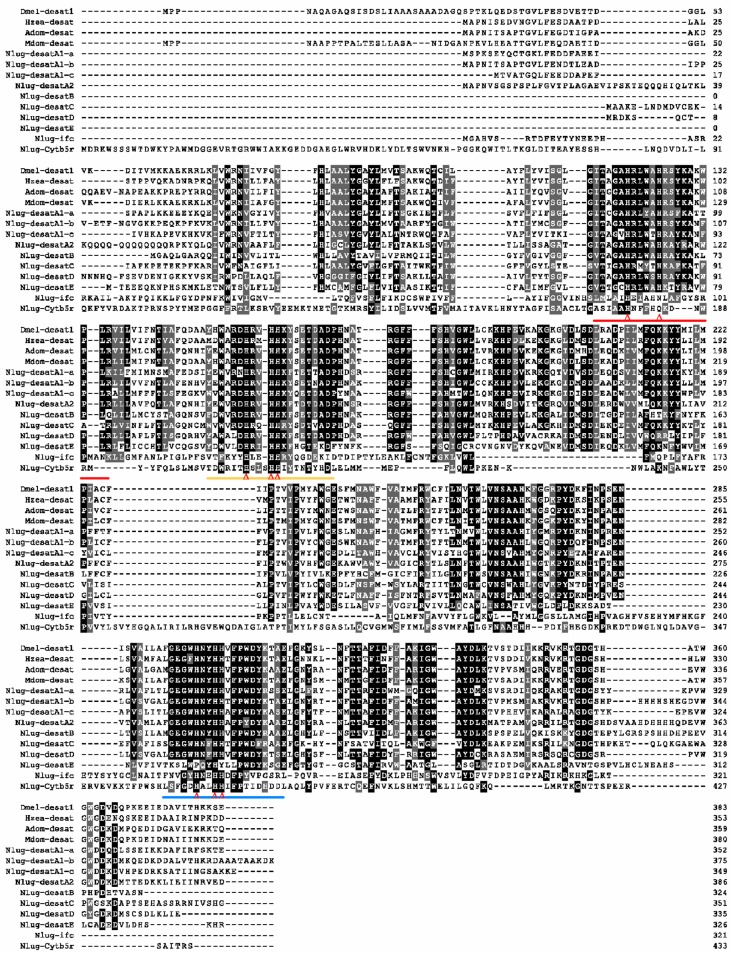
Amino acid sequence alignment of 10 *Nilaparvata lugens* desaturases with known desaturases. The colors of the aligned sequences showed the degree of amino acid similarity, including identical (black) and 70% similarity (dark grey). Three conserved histidine (His)-containing regions are in bold below the alignment (red, region Ⅰa; yellow, region Ⅰb; blue, region Ⅱ). Red triangles under sequences indicate conserved His residues. Selected protein accession numbers: Dmel-desat1 (*Drosophila melanogaster*, CAB52474), Hzea-desat (*Helicoverpa zea*, AAF81788), Adom-desat (*Acheta domesticus*, AAK25796), Mdom-desat (*Musca domestica*, AAN31393).

The predicted amino acid sequences were further aligned with known Δ9-desaturases from other insects to identify salient features. Seven putative Δ9-desaturases (Nlug-desatA1-a, Nlug-desatA1-b, Nlug-desatA1-c, Nlug-desatA2, Nlug-desatB, Nlug-desatC, and Nlug-desatD) share high similarity with Δ9-desaturases from other insects in the conserved histidine (His)-containing regions (region Ⅰa, region Ⅰb and region Ⅱ) which are crucial for catalytic activity (Figure 2) [9,33]. However, in Nlug-desatE, the 6th and 8th conserved His residues were replaced by proline (P) and tyrosine (Y), respectively. This indicates that Nlug-desatE may have lost the catalytic function as a desaturase.

### 2.2. Phylogenetic Analyses of Nlug-desat Genes

**Figure 3:**
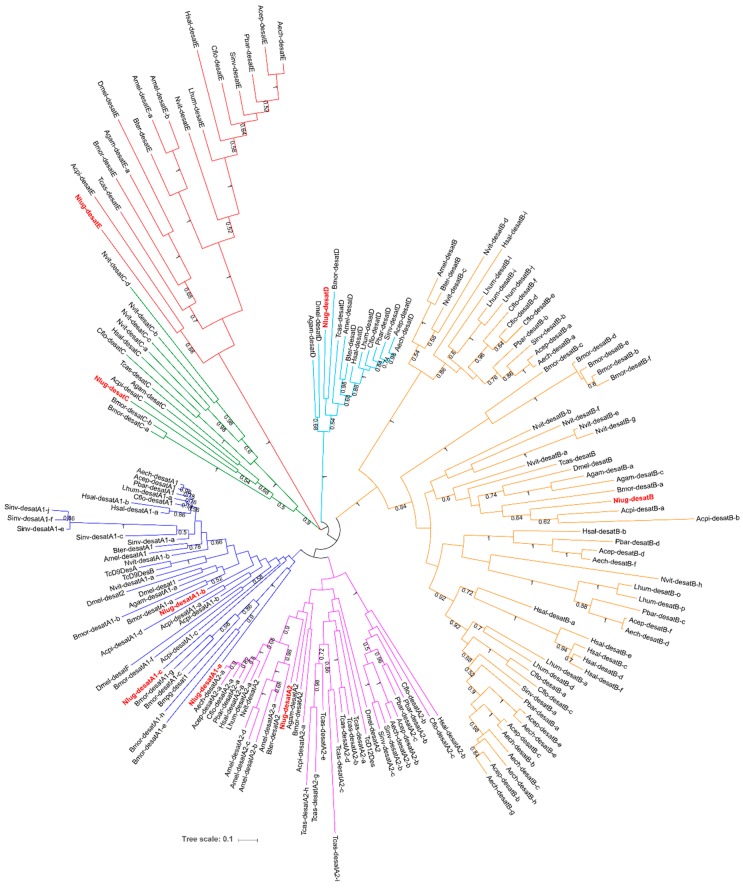
Maximum-likelihood phylogenetic tree of amino acid sequences of insect First Desaturase genes. The tree with the highest log likelihood (−58066.33) is shown. The proportion of trees in which the associated taxa clustered together in the bootstrap test (100 replicates) is shown next to the branches. There were a total of 311 positions in the final dataset derived from 177 genes of 16 species (Table S1). Gene names follow the updated nomenclature proposed by Helmkampf et al. (2015) [13], except for genes that have previously been characterized in the literature. Species are indicated by four-letter prefixes as follows: Aech, *Acromyrmex echinatior*; Acep, *Atta cephalotes*; Cflo, *Camponotus floridanus*; Hsal, *Harpegnathos saltator*; Lhum, *Linepithema humile*; Pbar, *Pogonomyrmex barbatus*, Sinv, *Solenopsis invicta*; Acpi, *Acyrthosiphon pisum*; Amel, *Apis mellifera*; Agam, *Anopheles gambiae*; Bmor, *Bombyx mori*; Bter, *Bombus terrestris*; Dmel, *Drosophila melanogaster*; Nvit, *Nasonia vitripennis*; Tcas, *Tribolium castaneum*; and Nlug, *Nilaparvata lugens* (in red). Branches of six subfamilies in the insect First Desaturase family are marked in different colors.

Phylogenetic construction revealed that eight members of the First Desaturase family in BPH can be clustered into six subfamilies (Desat A1, A2, B, C, D, and E). Three of them (Nlug-desatA1-a, Nlug-desatA1-b, and Nlug-desatA1-c) belong to the Desat A1 subfamily. Each of the other five subfamilies comprise a single-copy gene (Figure 3). On the other hand, BPH Cyt-b5-r (Nlug-Cytb5r) and Ifc (Nlug-ifc) bear little sequence similarity to the remaining Δ9-desaturases and each other (Figure 2). These two genes were, therefore, analyzed separately from the other eight putative desaturase genes in phylogenetic analyses [13]. The Cyt-b5-r group in BPH (Nlug-Cytb5r) possesses a single-copy gene, and clustered with the Cyt-b5-r genes in *Acyrthosiphon pisum* (Acpi-Cytb5r-a and Acpi-Cytb5r-b) and *Bombyx mori* (Bmor-Cytb5r-b) (Figure S1). Furthermore, Nlug-ifc (from BPH) clustered with the Ifc gene from *Acyrthosiphon pisum* (Figure S2).

### 2.3. Developmental Expression Patterns of Nlug-desat Genes

**Figure 4:**
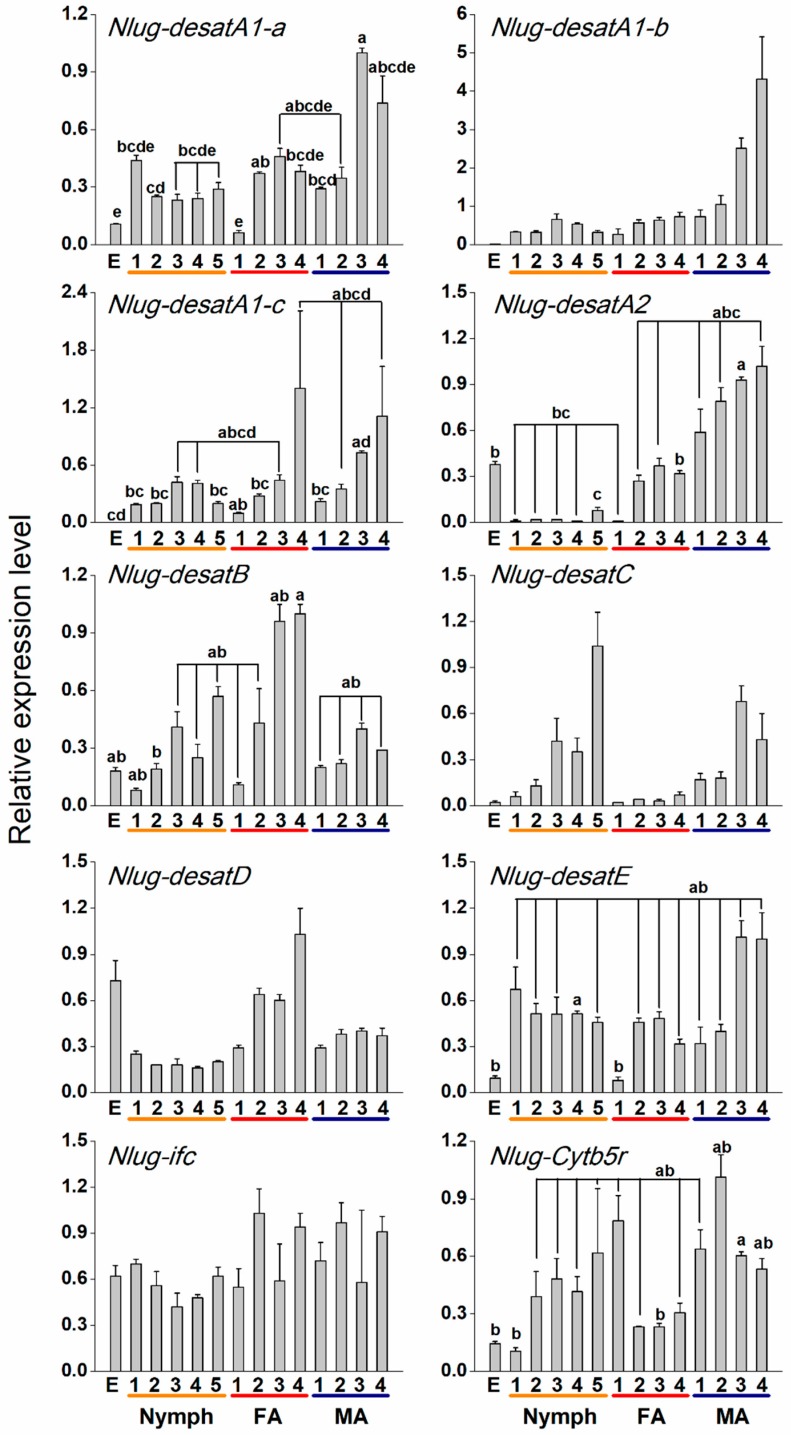
Mean transcript levels (+SE, *n* = 3) of 10 desaturase genes in different developmental stages of BPH. E, egg; Nymph 1–5, first- to fifth-instar larvae; FA 1–4, 1- to 4-d-old-female adult; MA 1–4, 1- to 4-d-old-male adult. The results (threshold cycle values) of the qRT-PCR assays were normalized to the expression level of *RPS15* (ribosomal protein S15e, GenBank accession number: ACN79501.1). Letters indicate significant differences among different treatments (*p* < 0.05). Statistical information is provided in Table S2 and Table S3.

The transcript level of each *Nlug-desat* gene varied with developmental stage, and each gene presented a specific developmental expression pattern. In general, *Nlug-desatA1-a* and *Nlug-desatE* showed low expression levels in egg and 1-d-old female, and their transcript levels peaked in 3- and 4-d-old males. *Nlug-desatA1-b*, *Nlug-desatD*, and *Nlug-ifc* had relatively constant expression levels at different developmental stages; meanwhile, the expression of *Nlug-desatA1-b* showed an increasing trend during the male adult stage. *Nlug-desatA1-c* had low expression levels at the egg stage but also showed high expression in the later adult stages with a considerable variation. *Nlug-desatA2* exhibits significantly lower expression levels during all five nymph stages compared to that in the 3-d-old males. The expression levels of *Nlug-desatB* peaked in the 4-d-old female. *Nlug-desatC* showed a rising trend of expression before emerging but with insignificant difference among stages. *Nlug-Cytb5r* had lower expression levels in the egg stage, first-instar larvae, and 3-d-old female compared with that in the 3-d-old male. (Figure 4 and Table S2).

### 2.4. Tissue-Specific Expression Patterns of Nlug-desat Genes

**Figure 5:**
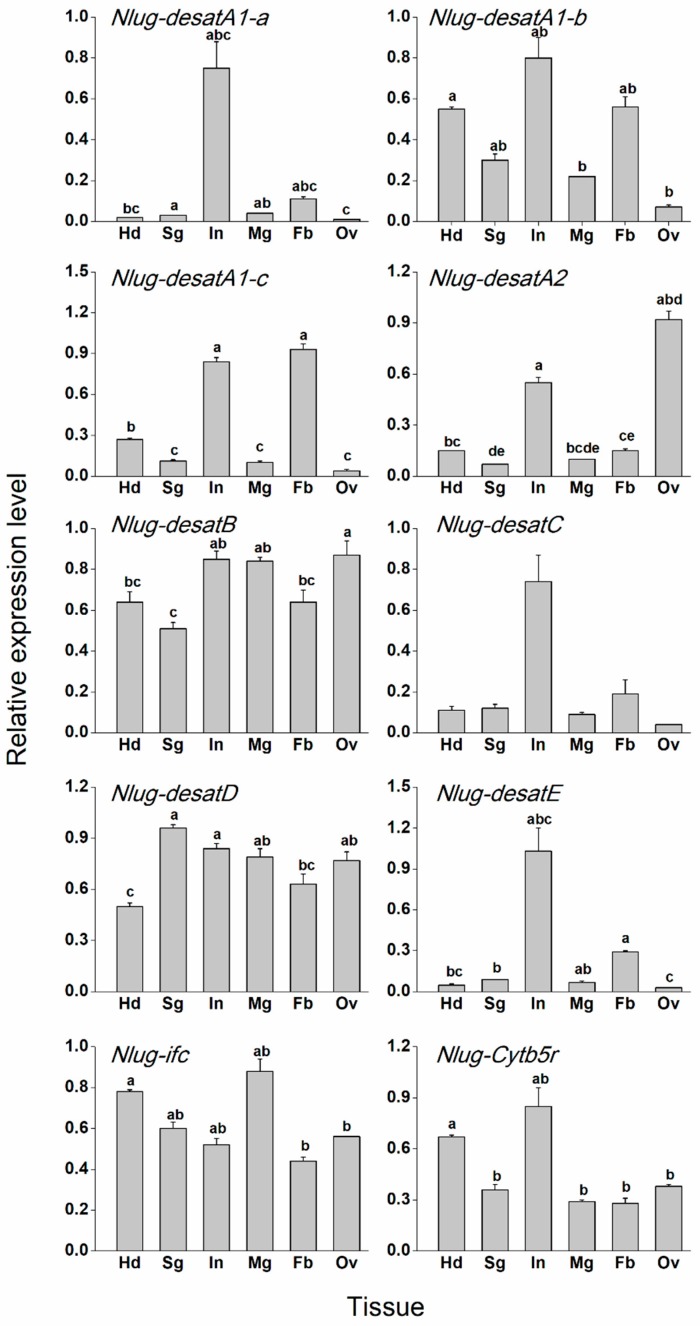
Mean transcript levels (+SE, *n* = 3) of 10 desaturase genes in different tissues of BPH. Hd, head; Sg, salivary gland; In, integument; Mg, midgut; Fb, body fat; Ov, ovary. All tissues were dissected from 4-d-old-female adults. The results (threshold cycle values) of the qRT-PCR assays were normalized to the expression level of *RPS11* (ribosomal protein S11, GenBank accession number: ACN79505.1). Letters indicate significant differences among different treatments (*p* < 0.05). Statistical information is provided in Table S4 and Table S5.

Tissue-specific expression pattern analysis revealed that *Nlug-desatA1-a*, *Nlug-desatC*, and *Nlug-desatE* had high but not significant expression in the integument. On the other hand, *Nlug-desatA1-b* showed a low expression level in the ovary. *Nlug-desatA1-c* was mainly expressed in the body fat and integument. *Nlug-desatA2* had a maximum expression in the ovary and integument. On the other hand, *Nlug-desatB*, *Nlug-desatD*, and *Nlug-ifc* only showed a slight variation of expression levels among different tissues. *Nlug-Cytb5r* had a relatively high expression level in the head (Figure 5 and Table S4).

### 2.5. Differences in Nlug-desat Genes Expression between TN1 and Babawee Populations

**Figure 6:**
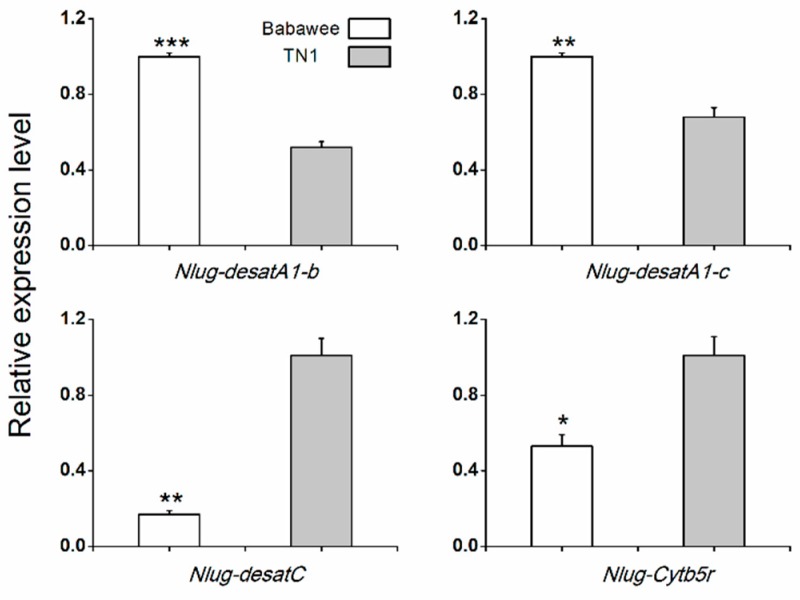
Mean transcript levels (+SE, *n* = 3) of 4 desaturase genes in BPH population reared on rice variety Babawee or TN1. The results (threshold cycle values) of the qRT-PCR assays were normalized to the expression level of *RPS15* (ribosomal protein S15e, GenBank accession number: ACN79501.1). Asterisks indicate significant difference between treatments (* *P* < 0.05, ** *P* < 0.01 and *** *P* < 0.001, Student’s *t* test).

To test for variation in expression of *Nlug-desat* genes with host variety, we compared the transcript levels of 10 *Nlug-desat* genes in 4-d-old female adults from populations of BPH reared on TN1 rice vs. Babawee rice using qRT-PCR methods. The mRNA levels of *Nlug-desatA1-b* and *Nlug-desatA1-c* were significantly higher in the whole body of Babawee-BPH compared with TN1-BPH. In contrast, *Nlug-desatC* and *Nlug-Cytb5r* had significantly higher expression levels in the whole body of TN1-BPH compared with Babawee-BPH (Figure 6). The transcript levels of the other 6 *Nlug-desat* genes were similar between two different populations (Supplementary Figure S3).

### 2.6. Influence of Nlug-desats Suppression on Fatty Acids of BPH Female Adults

**Figure 7:**
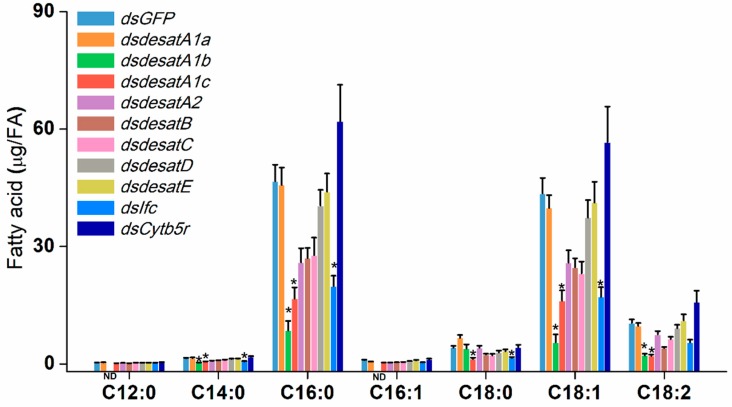
The effect of single knockdown of *Nlug-desat*s on fatty acids in BPH. Mean contents (+SE, *n* = 3) of fatty acids in the whole body of a 1-d-old-female BPH adult (FA) at 3 days after injection of the dsRNA of 10 *N. lugens* desaturase genes or *GFP* (*dsGFP*). **(A)** C12:0, lauric acid; C14:0, myristic acid; C16:0, palmitic acid; C16:1, palmitoleic acid; C18:0, stearic acid; C18:1, oleic acid; C18:2, linoleic acid. ND, no detection. Differences in specific fatty acid levels between the control group (dsGFP-BPH) and each treatment group were determined by Brown-Forsythe and Welch ANOVA followed by Dunnett’s T3 multiple comparisons test. Asterisks indicate significant difference between *dsGFP* injection and each *dsdesat* injection treatments (*p* < 0.05). Statistical information is provided in Table S6.

To investigate the possible involvement of *Nlug-desat*s in the BPH fatty acid metabolism, *Nlug-desat* genes used in this study were knocked down individually, and the fatty acids from each RNAi-treated BPH sample were analyzed. The dsRNA for each *Nlug-desat* gene was injected into the third-instar nymphs and the transcript levels of each target gene were efficiently suppressed by 77.7% to 98.0% at 3 days post injection (dpi) (Supplementary Figure S4). Comparing the presence of saturated as well as unsaturated fatty acids, we noticed four saturated fatty acids: lauric acid (C12:0), myristic acid (C14:0), palmitic acid (C16:0) and stearic acid (C18:0). Three unsaturated fatty acids; palmitoleic acid (C16:1), oleic acid (C18:1) and linoleic acid (C18:2), were identified in the female BPH with C16:0 and C18:1 as the main constituent composition. Compared to the fatty acid profiles in dsGFP-BPH, knockdown of *Nlug-desatA1-b* significantly reduced the levels of C14:0, C16:0, C18:1, and C18:2. Moreover, C12:0 and C16:1 were not found in *Nlug-desatA1-b*-silenced BPH, suggesting that these fatty acids may have been reduced as well. Knockdown of *Nlug-desatA1-c* significantly reduced all fatty acids besides C12:0 and C16:1. Knockdown of *Nlug-ifc* significantly reduced the levels of C14:0, C16:0, C18:0, and C18:1 (Figure 7 and Table S6). Compared with dsGFP-BPH, *Nlug-desatA1-b*-, *Nlug-desatA1-c*- and *Nlug-ifc*-silenced BPH, have significantly less saturated and unsaturated fatty acids. Oppositely, knockdown of any other *Nlug-desat*s do not influence the levels of all identified fatty acids (Figure 7 and Supplementary Figure S5). Compared with dsGFP-BPH, the ratio of C16:1/C16:0 was only significantly reduced in dsdesatA1-b-BPH. Knockdown of *Nlug-desatA1-a*, *Nlug-desatA1-b* and *Nlug-desatA2* reduced the ratio of C18:1/C18:0. Oppositely, *Nlug-desatE*- and *Nlug-Cytb5r*-silenced BPH have a higher ratio of C18:1/C18:0 (Supplementary Figure S6).

### 2.7. Knockdown of Nlug-desats Decrease the Survival Rate of BPH Nymphs

**Figure 8:**
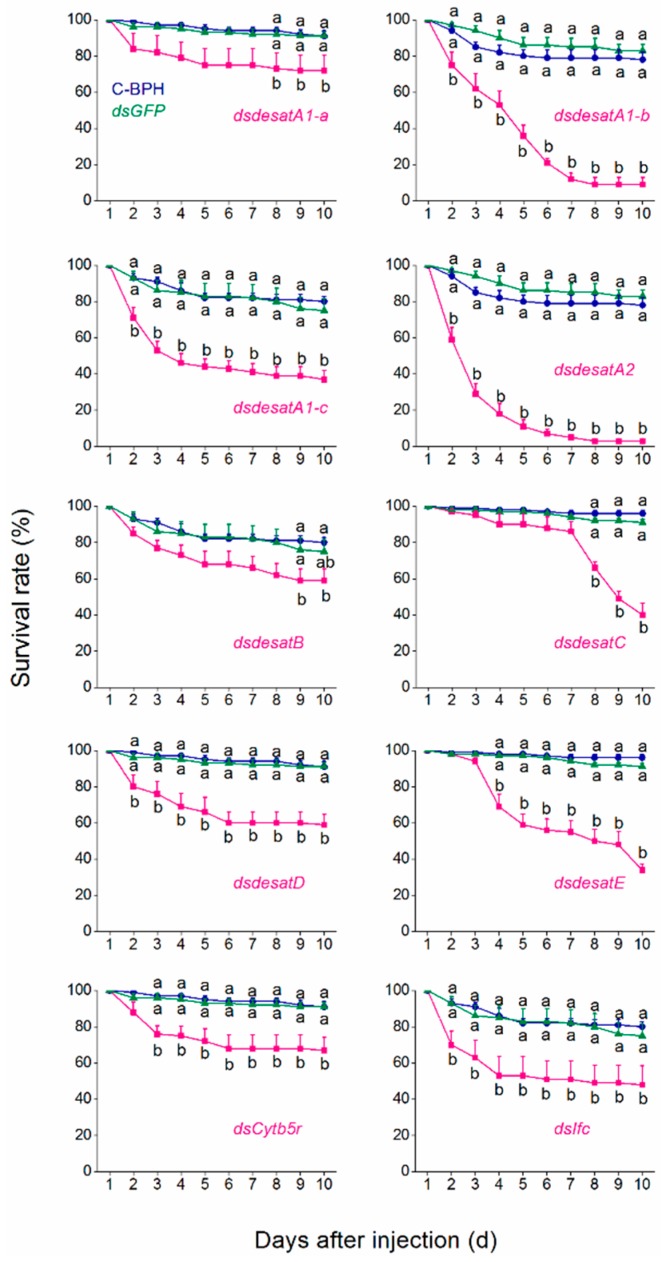
The effect of single knockdown of *Nlug-desat*s on BPH nymph survival. Mean survival rate (+SE, *n* = 5) of BPH injected with the dsRNA of 10 *N.lugens* desaturase genes or *GFP* (*dsGFP*), or kept non-injection (C-BPH). Letters indicate significant differences among different treatments (*p* < 0.05, one-way ANOVA followed by Duncan’s test).

We investigated whether knockdown of *Nlug-desat*s could influence the survival rate of BPH. Compared with dsGFP-BPH (BPH injected with the dsRNA of *GFP*) and C-BPH (non-injected BPH), BPH injected with dsRNAs of seven *Nlug-desats* (*Nlug-desatA1-b*, *Nlug-desatA1-c*, *Nlug-desatA2*, *Nlug-desatD*, *Nlug-desatE*, *Nlug-Cytb5r*, and *Nlug-Ifc*) separately exhibited a significantly lower survival rate 7 dpi. The survival rates of dsdesatA1-b-, dsdesatA1-c-, dsdesatA2-, and dsdesatE-BPH were lower than 40% at 10 dpi (9%, 37%, 3%, and 34%, respectively). Interestingly, compared with dsGFP-BPH and C-BPH, knockdown of *Nlug-desatC* did not affect the survival rate of BPH during 7 dpi, but the survival rate of dsdesatC-BPH rapidly declined from 8 dpi and dropped to 40% at 10 dpi. Of the ten desaturase genes, silencing of *Nlug-desatA2* resulted in the lowest survival rate of BPH (Figure 8).

## 3. Discussion

In this study, we identified and cloned 10 putative desaturase genes from the *N. lugens* transcriptome database. Phylogenetic analysis revealed that eight members of First Desaturase family genes in BPH could be clustered into five subfamilies (Figure 3). The Nlug-Cytb5r and Nlug-ifc belong to Cyt-b5-r [13] (Supplementary Figure S1) and infertile crescent (Ifc) protein, a Sphingolipid Desaturase with Δ4 activity [14] (Supplementary Figure S2), respectively.

Nlug-Cytb5r ortholog, commonly known as Cyt-b5-r, has reportedly been down-regulated in the activation of immune defense in honey bee larvae [15] and cold acclimatization of *Drosophila virilis* [16]. A low copy-number in the Cyt-b5-r subfamily of insects suggests their fundamental role in lipid metabolism [13]. The single-copy gene *Nlug-Cytb5r*, which had a minor variation of expression levels at developmental stages (Figure 4 and Table S2) or in different tissues (Figure 5 and Table S4), might serve as a housekeeping gene involved in lipid metabolism. However, knockdown of *Nlug-Cytb5r* did not significantly alter the fatty acid composition of BPH (Figure 7 and Supplementary Figure S5). These results suggest that *Nlug-Cytb5r* might not be essentially needed in the regulation of fatty acid levels during BPH development. However, the *Nlug-Cytb5r* gene might also be necessary for other essential metabolic functions in BPH. The expression of *Nlug-Cytb5r* might be stabilized within compensatory mechanisms past day 3 of RNAi, which might be sufficient to rescue the potential lethal phenotype of dsCytb5r-BPH. Previously, some beetle strains, such as *Tribolium castaneum* [42], *Rhyzopertha dominica* (F.) [43] and *Sitophilus oryzae* (L.) [44], lacking in functional copies of Cyt-b5-r, did not show adverse effects on development or reproduction under laboratory conditions [45]. It is also possible that other functional homologs of Cyt-b5-r gene, e.g., Cytochrome b5 (cyb5), can maintain the primary function of Cyt-b5-r as well as the survival of nymphs in the absence of Nlug-Cytb5r activity.

It has already been reported that Ifc protein is associated with meiosis [46] and thereby involved in the reproduction of *Drosophila* [18]. However, very little is on record regarding the involvement of Ifc protein in lipid metabolism. Similar to the Cyt-b5-r gene *Nlug-Cytb5r*, the Ifc gene *Nlug-Ifc* has a single-copy as well (Supplementary Figure S2). The constant mRNA level of *Nlug-ifc* during different developmental stages (Figure 4) and in different tissues (Figure 5) implies its crucial function in the lipid metabolism pathway. Additionally, knockdown of *Nlug-ifc* significantly down-regulated the composition of unsaturated and saturated fatty acids (Figure 7 and Supplementary Figure S5). In a recent report, disruption of fatty acid composition in *Drosophila melanogaster* larvae fed with SCD1 (also commonly known as Δ9 desaturases) inhibitor CAY10566 resulted in a lethal phenotype [22]. Here, we speculate that fatty acid deficiency caused by *Nlug-ifc*-knockdown might contribute to decreasing the survival rate of dsIfc-BPH (Figure 8).

In the present study, the size of the First Desaturase gene family in *N. lugens* (8 genes) is approximately similar to that in *A. pisum* (10 genes), *D. melanogaster* (7 genes) and *Apis mellifera* (9 genes). However, this is much smaller than the gene family size in *T. castaneum* (15 genes) or *Bombyx mori* (20 genes) [13]. Hahn et al. [47] attributed such differences in gene family size between genomes to adaptation. The replacement of conserved His residues in *Nlug-desatE* suggests the potential loss of desaturation activity (Figure 2). This is based upon the essential catalytic role of conserved histidine residues in the membrane-bound fatty acid desaturases [9,12]; however, additional research would be needed to verify its function. Interestingly, Nlug-desatE did not significantly affect fatty acid abundance or composition (Figure 7 and Supplementary Figure S5), but the survival rate of dsdesatE-BPH nymphs decreased significantly (Figure 8). These results combined with the high expression level of *Nlug-desatE* in the integument suggest the essential requirement of *Nlug-desatE* in basic metabolism excluding fatty acid desaturation during development.

The three most abundant fatty acids identified in BPH female adults are palmitic acid (C16:0), oleic acid (C18:1) and linoleic acid (C18:2) (Figure 7). This is consistent with previous research about BPH [41]. An important function of DESAT1 in *D. melanogaster* is C16:1 and C18:1 production by introducing a double bond in C16:0 and C18:0 [48]. The content of C18:0 in dsdesatA1-a-BPH is 1.59-fold higher than that in dsGFP-BPH (Figure 7), which suggests that Nlug-desatA1-a may be involved in the induction of an unsaturated bond in C18:0. Further, due to the tissue expression pattern of *Nlug-desatA1-a* (Figure 5), *Nlug-desatA1-a* might play a role in cuticular hydrocarbon production. However, the total contents of saturated fatty acids and unsaturated fatty acids were not altered in the dsdesatA1-a-BPH (Supplementary Figure S5). Moreover, *Nlug-desatA1-a* silencing had only a small effect on the survival rate of BPH (Figure 8). We are of the opinion that other Nlug-desats in BPH may maintain the content of fatty acids in the absence of Nlug-desatA1-a activity.

Metabolic deficit is one of the important reasons for the lethal phenotype of *DESAT1*-deficient or -silenced *D. melanogaster* larvae. However, artificial diet supplemented with unsaturated fatty acids can rescue the survival of larvae [49]. Knockdown of *Nlug-desatA1-b*, *Nlug-desatA1-c*, and *Nlug-Ifc* not only significantly down-regulated unsaturated and saturated fatty acid composition but also significantly reduced the survival rate of BPH (Figure 7 and Figure 8). So we suggest that these two desaturases play an essential role in lipid metabolism and thereby maintain the survival of BPH. The lethal phenotype in *Nlug-desatA1-b-*, *Nlug-desatA1-c-*, and *Nlug-Ifc-*knockdown BPH may be due to deficiency of unsaturated fatty acids (Figure 7 and Supplementary Figure S5). Furthermore, this lethal phenotype may have appeared partly due to effects on feeding behavior in the previously mentioned desaturase gene-knockdown BPH. Previous results have confirmed that the feeding behavior of *D. melanogaster* larvae can be blocked by using desaturase inhibitor [22]. Contrarily, knockdown of *Nlug-desatB* did not affect the unsaturated and saturated fatty acids (Figure 7), and only slightly decreased the survival rate of *Nlug-desatB*-knockdown BPH (Figure 8). However, the relative expression level of *Nlug-desatB* was higher in 4-d-old-female adults compared to second-instar nymphs (Figure 4) and showed a rising trend from 1- to 4-d-old females, suggesting that the *Nlug-desatB* may still play a role in the lipid metabolism of the adult female. Similar to *Nlug-desatB*, *Nlug-desatE* and *Nlug-Cytb5r*, single knockdown of *Nlug-desatA2*, *Nlug-desatC*, and *Nlug-desatD* does not affect the level of each single fatty acid composition (Figure 7) or the total content of saturated and unsaturated fatty acids (Supplementary Figure S5). However, silencing any of these genes individually increased the mortality of BPH nymphs (Figure 8). These genes may perform other crucial functions besides FA metabolism, or their roles in maintaining the dynamic balance of lipid metabolism are firmly related to the developmental stage and tissue of BPH. Furthermore, fatty acid analysis across tissues and lifecycles will be necessary to determine the role of these desaturase genes during the development of BPH.

Previously, high expression of desaturases has been reported in the generalist species *Spodoptera littoralis*, as well as the less polyphagous species *Spodoptera frugiperda* (rice strain), but not in *S. frugiperda* (corn strain) [50]. Desaturases may have a potential role in the insect-plant interaction and be involved in the adaptation of herbivores. Interestingly, the expression of some desaturase genes in BPH was also modulated by food source. Higher expression levels of *Nlug-desatA1-b* and *Nlug-desatA1-c* in Babawee-BPH suggest their probable involvement in the adaptation of BPH to the resistant rice variety Babawee. Conversely, significantly higher transcript levels of *Nlug-desatC* and *Nlug-Cytb5r* in TN1-BPH compared with Babawee-BPH suggest the acceleration of lipid metabolism in TN1-BPH (Figure 6).

## 4. Materials and methods

### 4.1. Insect Rearing and Plant Growth

BPHs were originally provided by the Chinese National Rice Research Institute, Hangzhou, Zhejiang, China, and maintained on fresh Taichun Native 1 (TN1) rice seedlings under controlled conditions (27 ± 1 °C, 70 ± 10% relative humidity and 14/10 h light/dark photoperiod). The Babawee-BPH for gene-expression analysis of two different BPH populations were maintained on Babawee, a rice variety containing the resistance gene *Bph4*, for more than 40 generations under the same controlled conditions of the TN1-BPH.

### 4.2. Identification and Amplification of Nlug-desat Genes

Ten desaturase genes characterized by a fatty acid desaturase type Ⅰ domain (InterPro ID: IPR005804) in *D. melanogaster* [51] were used to find potential Nlug-desat genes in BPH based on its fat body transcriptome database [41] by using BLAST search. The candidate Nlug-desat genes were confirmed by blastx search against the non-redundant protein sequences database (nr). Ten *Nlug-desat* genes were obtained by RT-PCR from total RNA isolated from 4-d-old brachypterous BPH females. The primers (Table S7) were designed on the basis of transcriptome data of BPH fat bodies [41]. The PCR products were cloned into the pMD19-T vector (TaKaRa) and sequenced. Sequences were deposited in GenBank with the accession numbers (MH271225-MH271234).

### 4.3. Sequence Analysis and Phylogenetic Construction

The open reading frame (ORF) of *Nlug-desat*s was predicted by using the ORF Finder (https://www.ncbi.nlm.nih.gov/orffinder/). Domains of Nlug-desats were searched using the Batch CD-search (https://www.ncbi.nlm.nih.gov/Structure/bwrpsb/bwrpsb.cgi) and InterProScan (http://www.ebi.ac.uk/interpro/search/sequence-search). Amino acid sequences of insect desaturase sequences downloaded from NCBI (National Center for Biotechnology Information, http://www.ncbi.nlm.nih.gov) were aligned using Clustal Omega (https://www.ebi.ac.uk/Tools/msa/clustalo/). The evolutionary history was inferred by using the Maximum Likelihood method based on the Le Gascuel (2008) model [52]. This method was chosen after carrying a best-fit substitution model analysis in MEGA7 [53]. The tree with the highest log likelihood (−58066.33) is shown. The proportion of trees in which the associated taxa clustered together in the bootstrap test (100 replicates) is shown next to the branches. Initial tree(s) for the heuristic search were obtained automatically by applying Neighbor-Join and BioNJ algorithms to a matrix of pairwise distances estimated using a JTT model, and then selecting the topology with superior log likelihood value. A discrete Gamma distribution was used to model evolutionary rate differences among sites (5 categories (+G, parameter = 0.8376)). The tree is drawn to scale, with branch lengths measured in the number of substitutions per site. The bar represents 0.1 amino acid substitutions per sequence position. There were a total of 311 positions (from 177 putatively functional genes) in the final dataset. Evolutionary analyses were conducted in MEGA7 [53]. Graphical representation of the phylogenetic tree were performed with Interactive Tree of Life (version 3.5.1) [54,55]. Phylogenetic analysis of Cyt-b5-r and Ifc were constructed as outlined above for Cyt-b5-r (425 amino acid positions derived from 26 putatively functional genes) and Ifc (321 amino acid positions from 18 putatively functional genes).

### 4.4. RNA Preparation and Real Time qPCR

Total RNA was extracted from (1) whole bodies of BPH at different developmental stages (eggs (*n* = 200), first-instar nymphs (*n* = 60), second-instar nymphs (*n* = 60), third-instar nymphs (*n* = 20), fourth-instar nymphs (*n* = 20), fifth-instar nymphs (*n* = 20), newly emerged to 4-d-old brachypterous male and female adults (*n* = 20)); (2) six specific BPH tissue samples (head, salivary gland, integument, midgut, fat body and ovary) dissected from 4-d-old brachypterous female adults (*n* = 200); (3) 4-d-old brachypterous female adults from TN1 population and Babawee popupaltion (*n* = 20). Total RNA was isolated using the SV Total RNA Isolation System (Promega) according to the manufacturer’s instructions. Each total RNA sample (500 ng) was reverse transcribed using the PrimeScript™ RT reagent kit (TaKaRa). Real time qPCR was performed on the CFX96™ Real-Time system (Bio-Rad, Hercules, CA, USA) using iQ SYBRGreen Supermix (Bio-rad). The PCR program was 95 °C (3 min), followed by 40 cycles at 95 °C (15 sec) and 60 °C (20 sec). *RPS15* (ribosomal protein S15e, GenBank accession number: ACN79501.1) and *RPS11* (ribosomal protein S11, GenBank accession number: ACN79505.1) were used as reference genes in the analyses of developmental stage and tissue-specific expression patterns, respectively [56]. Primers used for real time qPCR were designed with PrimerPremier 5 (Table S7). Three independent biological replicates were analyzed, after which the average threshold cycle (C_t_) per sample was calculated. For the tissue-specific and developmental expression analysis of each desaturase gene, the sample with the lowest C_t_ value was designated as the calibrator. Tissue samples were chosen as follows: *Nlug-desatA1-a*, *Nlug-desatA1-b*, *Nlug-desatC*, *Nlug-desatE*, and *Nlug-Cytb5r*, integument; *Nlug-desatA2* and *Nlug-desatB*, ovary; *Nlug-desatD*, salivary gland; *Nlug-Ifc*, midgut; *Nlug-A1-c*, fat body. Developmental samples used as calibrators: *Nlug-desatA1-a* and *Nlug-desatE*, 3-d-old-male adult; *Nlug-desatA2*, 4-d-old-male adult; *Nlug-desatA1-c*, *Nlug-desatB*, and *Nlug-desatD*, 4-d-old-female adult; *Nlug-desatC*, five-instar larvae; *Nlug-Ifc*, 2-d-old-female adult; *Nlug-Cytb5r* and *Nlug-desatA1-b*, 2-d-old-male adult (the relative expression level of *Nlug-desatA1-b* in the egg stage will close to zero if 4-d-old-male adult was chosen as the calibrator). The relative expression levels were calculated using the 2^-△△Ct^ method.

### 4.5. RNAi Experiment

A unique region of each *Nlug-desat* gene was amplified by RT-PCR with primers including a T7 promoter sequence (Table S7). The purified PCR products were used to synthesize dsRNAs by using MEGAscript T7 High Yield Transcription Kit (Ambion, Austin, TX, USA). Third or fifth-instar nymphs were injected by using the FemtoJet (Eppendorf, Hamburg, Germany) microinjection device [57]. Each nymph was injected with about 0.25 μg of dsRNA of individual *Nlug-desat* or *GFP* (dsGFP, control) or not injected (C-BPH, control). The levels of *Nlug-desat* transcripts in the whole body of injected and control samples (third-instar nymphs were injected) were investigated 3 dpi. Individual nymphs were pooled (*n* = 20) for RNA extraction. The results (threshold cycle values) of the qRT-PCR assays were normalized to the expression level of *RPS15*. Three replicates were analyzed.

Due to the massive consumption of BPH in bioassay, the survival rates of BPH were investigated in four independent experiments on a different date. The *Nlug-desatA1-a-*, *Nlug-desatD-*, and *Nlug-Cytb5r*-silenced BPH were used in experiment 1; the *Nlug-desatC-* and *Nlug-desatE*-silenced BPH were used in experiment 2; the *Nlug-desatA1-c-*, *Nlug-desatB-*, and *Nlug-ifc-*silenced BPH were used in experiment 3; and the *Nlug-desatA1-b*- and *Nlug-desatA2-*silenced BPH were used in experiment 4. Each experiment contained a dsGFP-BPH group and a C-BPH group as controls. Twenty insects were used for each treatment, and each treatment was performed five times in parallel. The number of surviving BPH nymphs was recorded every day.

### 4.6. Fatty Acid Analysis

Total lipids were extracted from groups of BPH female adults at 3 dpi of *Nlug-desat*s or *GFP* (fifth-instar nymphs were injected). The BPH samples (9 female adults) were homogenized in 600 μL petroleum ether using a Precellys 24 homogenizer (Bertin) and centrifuged at 10,000 × g for 5 min at 4 °C. The supernatant was transferred into pre-weighed centrifuge tubes, evaporated by a rotavapor till dried and re-weighed.

Fatty acid concentrations were measured by forming fatty acid methyl esters (FAMEs) as described by Li et al. [58]. Ten microliters of methyl heptadecanoate (4 μg/μL) was added into 100 μL supernatant as internal standard. FAMEs were analyzed by using Agilent GC6890 gas chromatography equipped with a flame ionization detector (280 °C) through Agilent DB-23 column (60-m × 0.25-mm id 0.15-μm). Chromatography conditions: 250 °C inlet temperature, 242.3 kPa, splitless; 1 μL injection volume; N_2_ carrier gas (30 mL/min); H_2_ flow 40 mL/min, air flow 445 mL/min; the oven temperature was held at 50 °C for 1 min, increased to 175 °C at 25 °C /min, then at 3.5 °C /min to 230 °C and finally held at 230 °C for 5 min. FAMEs were identified by comparison with the Supelco^®^ 37 Component FAME Mix. The fatty acid contents were calculated based on three experiments.

### 4.7. Data Analysis

The statistical analyses were conducted by using the PASW^®^ Statistics 18 and Prism 8.0.2 software. Developmental and tissue-specific gene expression patterns were analyzed by using Brown-Forsythe and Welch ANOVA followed by Tamhane’s T2 multiple comparisons test. Mean transcript levels of *Nlug-desatB* and *Nlug-desatD* in different tissues of BPH were analyzed by using ordinary one-way ANOVA followed by Tukey’s test. For fatty acid analysis, differences in specific fatty acid levels between a control group (dsGFP-BPH) and each treatment group were determined by Brown-Forsythe and Welch ANOVA followed by Dunnett’s T3 multiple comparisons test. Due to the undetectable levels of C12:0 and C16:1 in dsdesatA1-b-BPH, they were excluded from the multiple comparisons, and differences in the ratio of C16:1/C16:0 between dsdesatA1-b-BPH and dsGFP-BPH was analyzed by using *t*-test with Welch’s correction. Differences in BPH survival rate between treatments and silencing efficiency of BPH desaturase genes were determined by one-way ANOVA followed by Duncan’s test. Student’s *t*-test for comparing differences in *Nlug-desat* genes expression between TN1 and Babawee populations.

## 5. Conclusions

We have identified and annotated the acyl-CoA desaturase gene family in BPH, which contains eight First Desaturases, one sphingolipid delta-4 desaturase, and one cytochrome-b5-related desaturase. Results have confirmed the role of *Nlug-desat*s in lipid metabolism and survival of BPH upon different rice varieties. These findings are of paramount significance with respect to BPH desaturases and offer aid in developing novel strategies for pest control.

## Supplementary Materials

Supplementary materials can be found at https://www.mdpi.com/1422-0067/20/6/1369/s1.
