# Supplementary material for: The Desaturase Gene Family is Crucially Required for Fatty Acid Metabolism and Survival of the Brown Planthopper, Nilaparvata lugens
Source: Int J Mol Sci. 2019 Mar 19;20(6):1369. doi: 10.3390/ijms20061369 (PMC6472150; doi:10.3390/ijms20061369)
Supplement: Supplementary file 1 [file ijms-20-01369-s001.zip › ijms-447697-supplementary/ijms-447697-supplementary.pdf]

The desaturase gene family is crucially required for fatty acid metabolism and survival of the brown planthopper, *Nilaparvata lugens*

Jia-mei Zeng, Wen-feng Ye, Ali Noman, Ricardo A.R. Machado and Yong-gen Lou *

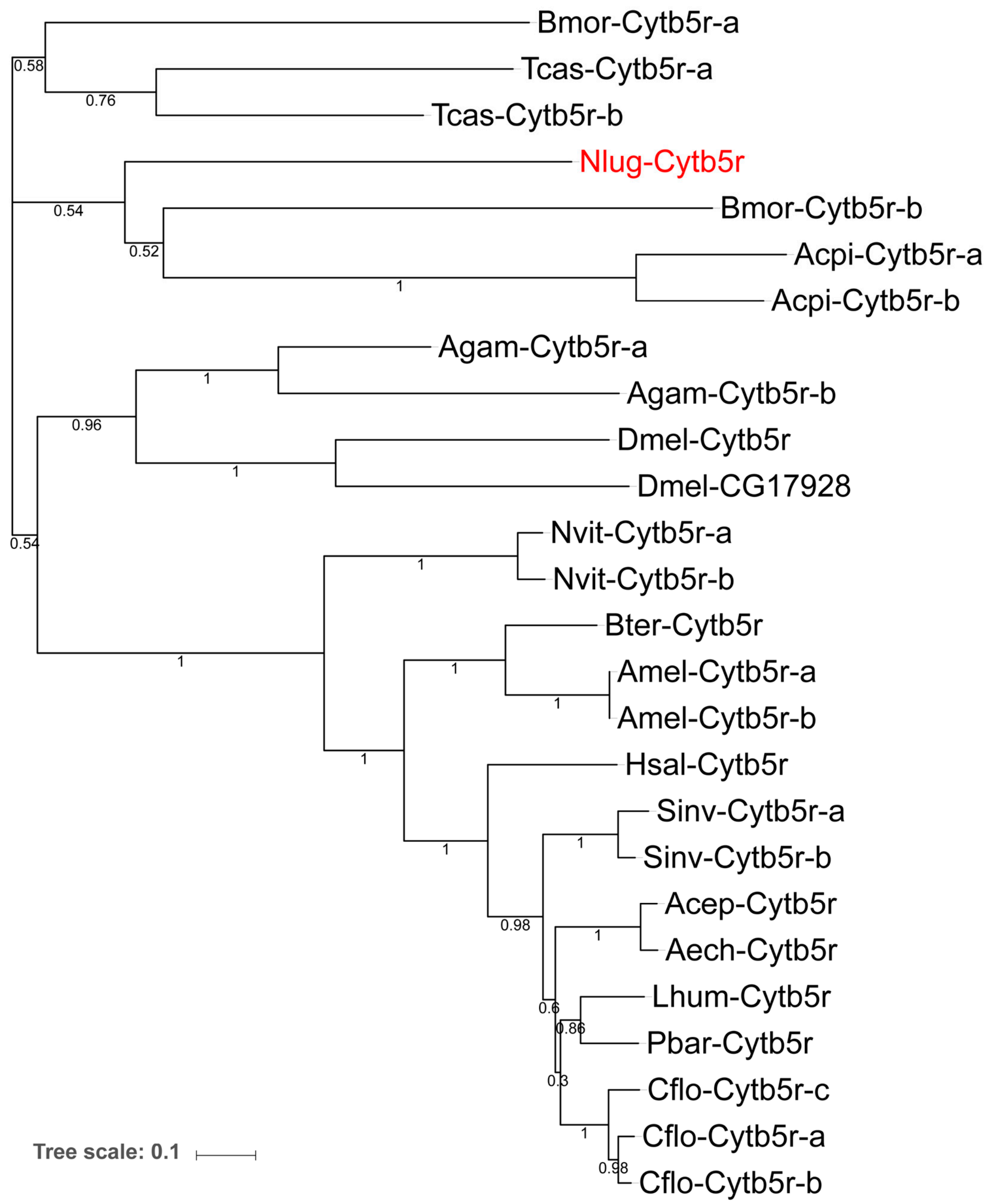

Supplemental Figure S1. Phylogenetic tree of insect cytochrome b5 fused desaturase genes (subfamily Cyt-b5-r) from 26 genes of 16 species.

The evolutionary history was inferred by using the Maximum Likelihood method model. The tree with the highest log likelihood (-13066.96) is shown. The proportion of trees in which the associated taxa clustered together is shown next to the branches. Initial tree(s) for the heuristic search were obtained automatically by applying Neighbor-Join and BioNJ algorithms to a matrix of pairwise distances estimated using a JTT model, and then selecting the topology with superior log likelihood value. A discrete Gamma distribution was used to model evolutionary rate differences among

sites (5 categories (+G, parameter = 1.6676)). The rate variation model allowed for some sites to be evolutionarily invariable ([+I], 8.62% sites). The tree is drawn to scale, with branch lengths measured in the number of substitutions per site. Bar represents 0.1 amino acid substitutions per sequence position. There were a total of 425 positions in the final dataset. Gene names follow the nomenclature based on the previously characterized *Cyt-b5-r* gene of *D. melanogaster*. Species are indicated by four-letter prefixes as follows: Aech = *Acromyrmex echinatior*, Acep = *Atta cephalotes*, Cflo = *Camponotus floridanus*, Hsal = *Harpegnathos saltator*, Lhum = *Linepithema humile*, Pbar = *Pogonomyrmex barbatus*, Sinv = *Solenopsis invicta*, Acpi = *Acyrthosiphon pisum*, Amel = *Apis mellifera*, Agam = *Anopheles gambiae*, Bmor = *Bombyx mori*, Bter = *Bombus terrestris*, Dmel = *Drosophila melanogaster*, Nvit = *Nasonia vitripennis*, Tcas = *Tribolium castaneum*, and Nlug = *Nilaparvata lugens* (marked in red, accession number MH271225).

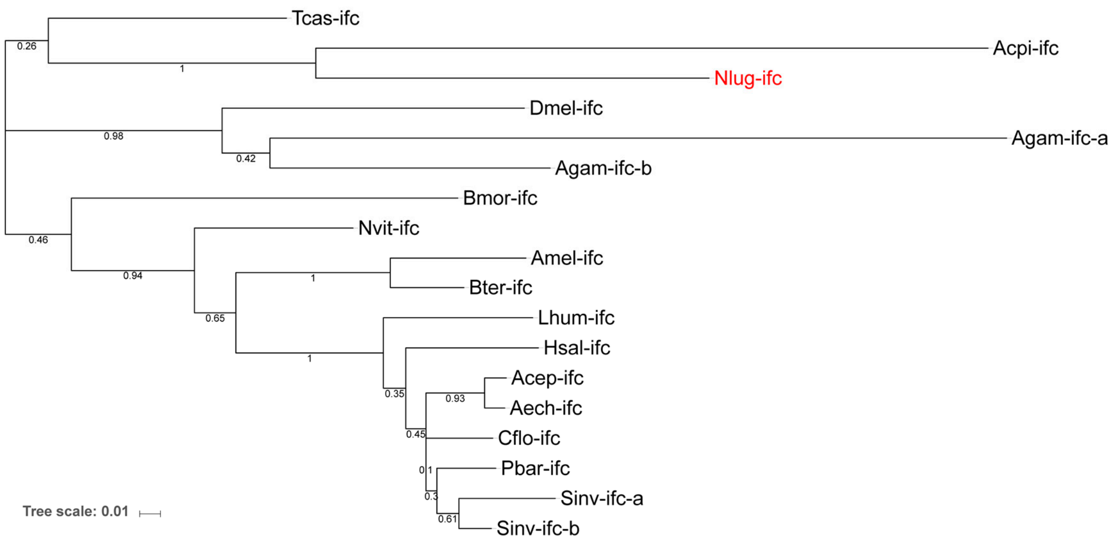

Supplemental Figure S2. Phylogenetic tree of Sphingolipid Desaturase genes (Ifc subfamily) from 18 genes of 16 species.

The evolutionary history was inferred by using the Maximum Likelihood method model. The tree with the highest log likelihood (-3900.66) is shown. The proportion of trees in which the associated taxa clustered together is shown next to the branches. Initial tree(s) for the heuristic search were obtained automatically by applying Neighbor-Join and BioNJ algorithms to a matrix of pairwise distances estimated using a JTT model, and then selecting the topology with superior log likelihood value. A discrete Gamma distribution was used to model evolutionary rate differences among sites (5 categories (+G, parameter = 0.4771)). The tree is drawn to scale, with branch lengths measured in the number of substitutions per site. Bar represents 0.01 amino acid substitutions per sequence position. There were a total of 321 positions in the final dataset. Gene names follow the nomenclature based on the previously characterized ifc gene of *D. melanogaster*. Species are indicated by four-letter prefixes as follows: Aech = *Acromyrmex echinatior*, Acep = *Atta cephalotes*, Cflo = *Camponotus floridanus*, Hsal = *Harpegnathos saltator*, Lhum = *Linepithema humile*, Pbar = *Pogonomyrmex barbatus*, Sinv = *Solenopsis invicta*, Acpi = *Acyrthosiphon pisum*, Amel = *Apis mellifera*, Agam = *Anopheles gambiae*, Bmor = *Bombyx mori*, Bter = *Bombus terrestris*, Dmel = *Drosophila melanogaster*, Nvit = *Nasonia vitripennis*, Tcas = *Tribolium castaneum*, and Nlug = *Nilaparvata lugens* (marked in red, accession number MH271230).

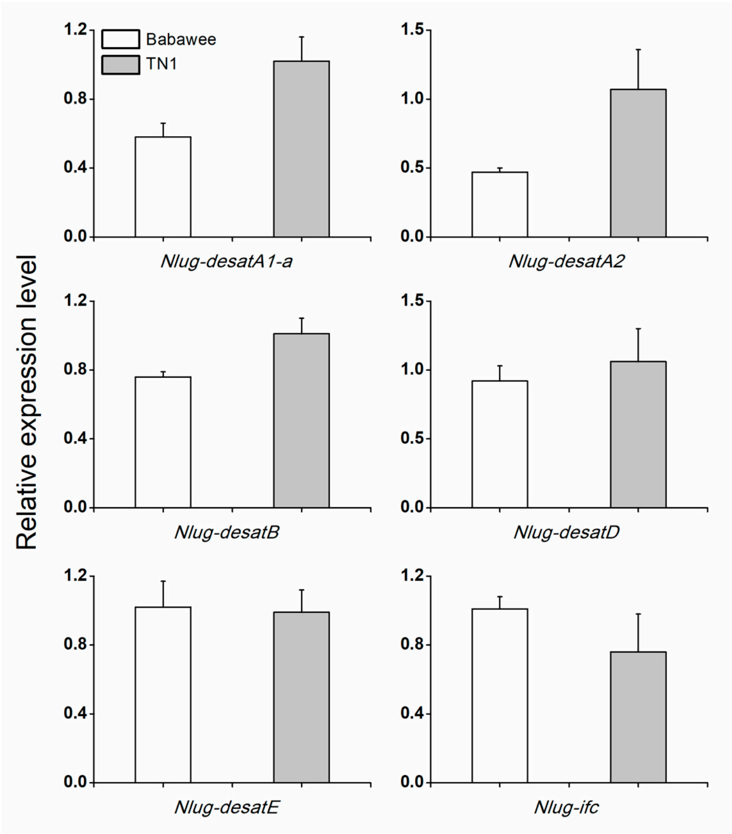

Supplemental Figure S3. Mean transcript levels (+SE, *n* = 3) of 6 desaturase genes in BPH population reared on rice variety Babawee or TN1.

The results (threshold cycle values) of the qRT-PCR assays were normalized to the expression level of *RPS15* (ribosomal protein S15e, GenBank accession number: ACN79501.1). No significant difference between treatments is found (Student's *t* test).

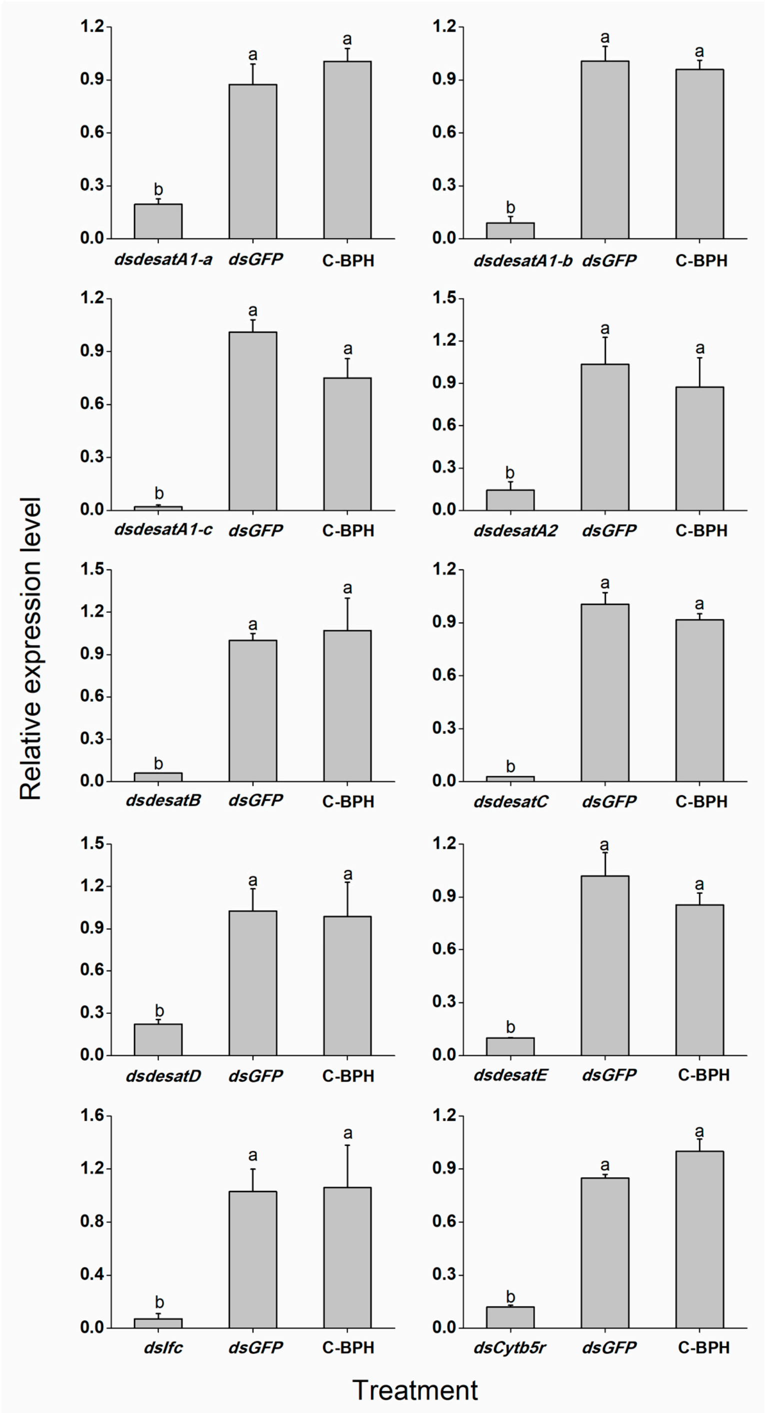

Supplemental Figure S4. The silencing efficiency of 10 *Nilaparvata lugens* desaturase genes by RNAi.

Mean transcript levels (+SE, $n = 3$) of *Nlug-desat*s in whole bodies on the third day after third-instar BPH nymphs had been singly injected with dsRNA of *Nlug-desat*s or GFP (*dsGFP*), or kept non-injected (C-BPH). The results (threshold cycle values) of the qRT-PCR assays were normalized to the expression of *RPS15*. Letters indicate significant differences among different treatments ($p < 0.05$, Duncan's test).

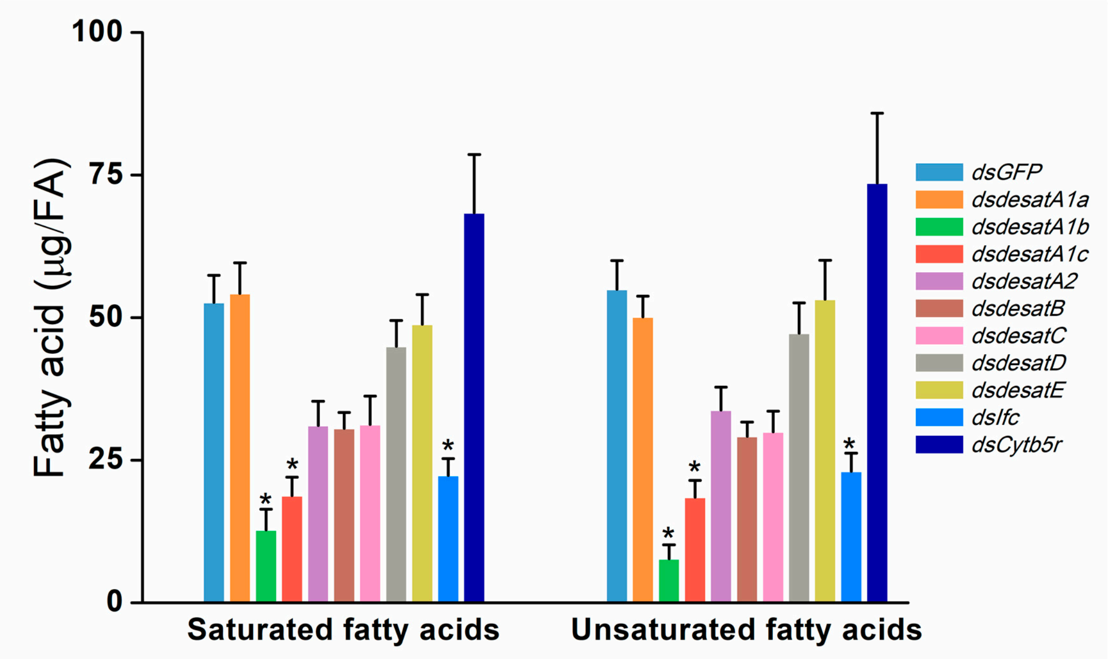

Supplemental Figure S5. The effect of single knockdown of *Nlug-desat*s on the level of total saturated and unsaturated fatty acids.

Mean contents (+SE, n = 3) of fatty acids in whole body of 1-d-old-female BPH adult (FA) at 3 days after injection of the dsRNA of 10 *N. lugens* desaturase genes or *GFP* (*dsGFP*). Differences in total fatty acid levels between the control group (dsGFP-BPH) and each treatment group were determined by Brown-Forsythe and Welch ANOVA followed by Dunnett's T3 multiple comparisons test. Asterisks indicate significant difference between dsGFP injection and each dsdesat injection treatments ($P < 0.05$).

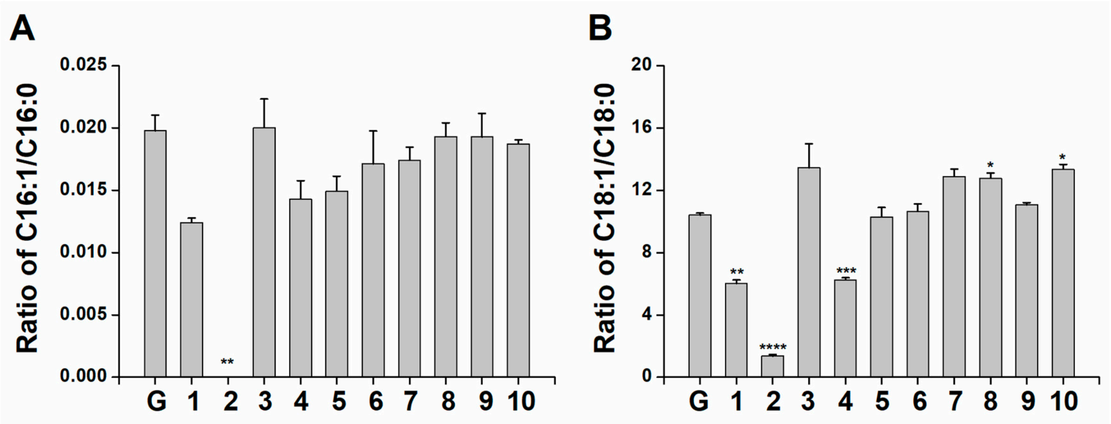

Supplemental Figure S6. The effect of single knockdown of *Nlug-desat*s on the ratio of C16:1/C16:0 and C18:1/C18:0 in BPH.

The ratio of C16:1/C16:0 (**A**) and C18:1/C18:0 (**B**) in the whole body of 1-d-old-female BPH adult at 3 days after injection of the dsRNA of 10 *N.lugens* desaturase genes or *GFP* (*dsGFP*). G, dsGFP-BPH; dsRNA of 1 to 10: 1, *Nlug-desatA1-a*; 2, Nlug-desatA1-b; 3, *Nlug-desatA1-c*; 4, *Nlug-desatA2*; 5, *Nlug-desatB*; 6, *Nlug-desatC*; 7, *Nlug-desatD*; 8, *Nlug-desatE*; 9, *Nlug-Ifc*; 10, *Nlug-Cytb5r*. Differences between a control group (dsGFP-BPH) and each treatment group were determined by Brown-Forsythe and Welch ANOVA followed by Dunnett's T3 multiple comparisons test. Due to the undetectable levels of C12:0 and C16:1 in dsdesatA1-b-BPH, they were excluded from the multiple comparisons, and differences in the ratio of C16:1/C16:0 between dsdesatA1-b-BPH and dsGFP-BPH was analyzed by using t-test with Welch's correction. Asterisks indicate significant difference between dsGFP injection and each dsNlDESAT injection treatments (*, P < 0.05; **, P < 0.01; ***, P < 0.001; ****, P < 0.0001).

Supplemental Table S2. Differences in the transcript levels of single *Nlug-desat* gene among developmental stages.

Gene ID	Tamhane's T2 multiple comparisons test	Mean Diff.	95.00% CI of diff.	Summary	Adjusted P Value
Nlug-desatA1-a	egg vs. 2nd	-0.1433	-0.2767 to -0.009825	*	0.0398
	egg vs. F2	-0.266	-0.3512 to -0.1808	***	0.0006
	egg vs. M1	-0.1844	-0.3177 to -0.05111	*	0.0175
	egg vs. M3	-0.8941	-1.722 to -0.06608	*	0.0425
	1st vs. M3	-0.5611	-0.9354 to -0.1867	*	0.0106
	2nd vs. F1	0.1873	0.02632 to 0.3483	*	0.0292
	2nd vs. F2	-0.1228	-0.2445 to -0.0009930	*	0.0486
	2nd vs. M3	-0.7508	-1.369 to -0.1323	*	0.0313
	3rd vs. M3	-0.7674	-1.153 to -0.3814	**	0.0036
	4th vs. M3	-0.7594	-1.136 to -0.3824	**	0.0034
	5th vs. M3	-0.7106	-1.179 to -0.2425	*	0.0112
	F1 vs. F2	-0.3101	-0.5077 to -0.1124	*	0.0129
	F1 vs. M1	-0.2284	-0.3894 to -0.06739	*	0.0143
	F1 vs. M3	-0.9381	-1.420 to -0.4564	**	0.0078
	F4 vs. M3	-0.6188	-1.076 to -0.1614	*	0.0169
	M1 vs. M3	-0.7097	-1.329 to -0.09084	*	0.0359
Nlug-desatA1-c	egg vs. F1	-0.09667	-0.1898 to -0.003566	*	0.0445
	1st vs. M3	-0.5433	-0.8930 to -0.1936	*	0.0117
	2nd vs. M3	-0.5333	-0.9393 to -0.1274	*	0.0224
	5th vs. M3	-0.5233	-0.8372 to -0.2095	**	0.0071
	F2 vs. M3	-0.4533	-0.8030 to -0.1036	*	0.0214
	M1 vs. M3	-0.51	-0.9034 to -0.1166	*	0.0193
Nlug-desatA2	egg vs. 5th	0.3009	0.05162 to 0.5501	*	0.0249
	egg vs. M3	-0.5514	-0.8061 to -0.2968	**	0.0025
	1st vs. M3	-0.9174	-1.378 to -0.4571	**	0.0096
	2nd vs. M3	-0.9144	-1.597 to -0.2318	*	0.0277
	3rd vs. M3	-0.9152	-1.581 to -0.2497	*	0.0261
	4th vs. M3	-0.9179	-1.600 to -0.2362	*	0.0274
	5th vs. F4	-0.2433	-0.4586 to -0.02802	*	0.0314
	5th vs. M3	-0.8523	-1.091 to -0.6136	***	0.0004
	F1 vs. M3	-0.9205	-1.654 to -0.1868	*	0.032
	F4 vs. M3	-0.609	-0.8478 to -0.3701	**	0.0014
Nlug-desatB	2nd vs. F4	-0.8167	-1.575 to -0.05861	*	0.0395
Nlug-desatE	egg vs. 4th	-0.4206	-0.6837 to -0.1575	**	0.0087
	4th vs. F1	0.4339	0.1408 to 0.7269	*	0.0112
Nlug-Cytb5r	egg vs. M3	-0.4575	-0.8461 to -0.06891	*	0.0314
	1st vs. M3	-0.4969	-0.7858 to -0.2080	**	0.0065
	F3 vs. M3	-0.3704	-0.6609 to -0.08003	*	0.0198

Differences in gene expression between different developmental stage were determined by Brown-Forsythe and Welch ANOVA followed by Tamhane’s T2 multiple comparisons test. 1th to 5th, first- to fifth-instar larvae; F1-4, 1- to 4-d-old-female adult; M1-4, 1- to 4-d-old-male adult.

Supplemental Table S4. Differences in the transcript levels of single *Nlug-desat* gene among tissues.

Gene ID	Tamhane's T2 multiple comparisons test	Mean Diff.	95.00% CI of diff.	Summary	Adjusted P Value
Nlug-desatA1-a	Hd vs. Sg	-0.00992	-0.01695 to -0.002892	*	0.0164
	Sg vs. Ov	0.01612	0.007082 to 0.02516	**	0.009
	Mg vs. Ov	0.02553	0.006420 to 0.04463	*	0.0221
Nlug-desatA1-b	Hd vs. Mg	0.32	0.2190 to 0.4210	**	0.0027
	Hd vs. Ov	0.4733	0.3447 to 0.6020	***	0.0007
Nlug-desatA1-c	Hd vs. Sg	0.1533	0.07983 to 0.2268	**	0.0036
	Hd vs. In	-0.5733	-0.8733 to -0.2733	*	0.0103
	Hd vs. Mg	0.1667	0.09077 to 0.2426	**	0.0036
	Hd vs. Fb	-0.66	-1.174 to -0.1456	*	0.0291
	Hd vs. Ov	0.23	0.1294 to 0.3306	**	0.0027
	Sg vs. In	-0.7267	-1.068 to -0.3858	**	0.0092
	Sg vs. Fb	-0.8133	-1.369 to -0.2576	*	0.0226
	In vs. Mg	0.74	0.3814 to 1.099	*	0.0104
	In vs. Ov	0.8033	0.5512 to 1.055	**	0.0019
	Mg vs. Fb	-0.8267	-1.398 to -0.2552	*	0.0234
	Fb vs. Ov	0.89	0.4378 to 1.342	**	0.01
Nlug-desatA2	Hd vs. Sg	0.08	0.02926 to 0.1307	**	0.0091
	Hd vs. In	-0.4033	-0.7275 to -0.07917	*	0.031
	Sg vs. In	-0.4833	-0.8075 to -0.1592	*	0.0208
	In vs. Mg	0.45	0.08072 to 0.8193	*	0.0335
	In vs. Fb	0.4	0.1759 to 0.6241	**	0.0093
	Fb vs. Ov	-0.7667	-1.523 to -0.01033	*	0.0486
Nlug-desatE	Hd vs. Fb	-0.2433	-0.3703 to -0.1163	**	0.0048
	Sg vs. Fb	-0.2	-0.3749 to -0.02514	*	0.0359
	Sg vs. Ov	0.06333	0.01104 to 0.1156	*	0.0279
	Mg vs. Ov	0.04667	0.01737 to 0.07596	**	0.0087
	Fb vs. Ov	0.2633	0.04234 to 0.4843	*	0.0344
Nlug-ifc	Hd vs. Fb	0.3333	0.1756 to 0.4911	**	0.0055
	Hd vs. Ov	0.22	0.1190 to 0.3210	**	0.0071
Nlug-Cytb5r	Hd vs. Sg	0.3133	0.02029 to 0.6064	*	0.0421
	Hd vs. Mg	0.3833	0.2796 to 0.4870	***	0.0003
	Hd vs. Fb	0.39	0.06982 to 0.7102	*	0.0303
	Hd vs. Ov	0.29	0.1773 to 0.4027	**	0.0033
Gene ID	Tukey's multiple comparisons test	Mean Diff.	95.00% CI of diff.	Summary	Adjusted P Value
Nlug-desatB	Hd vs. Ov	-0.2333	-0.4586 to -0.008103	*	0.0408
	Sg vs. In	-0.33	-0.5552 to -0.1048	**	0.0037
	Sg vs. Mg	-0.33	-0.5552 to -0.1048	**	0.0037
	Sg vs. Ov	-0.3567	-0.5819 to -0.1314	**	0.0019
	Fb vs. Ov	-0.2333	-0.4586 to -0.008103	*	0.0408
Nlug-desatD	Hd vs. Sg	-0.4667	-0.6686 to -0.2647	****	<0.0001
	Hd vs. In	-0.3433	-0.5453 to -0.1414	**	0.0011
	Hd vs. Mg	-0.2967	-0.4986 to -0.09472	**	0.0036
	Hd vs. Ov	-0.27	-0.4719 to -0.06805	**	0.0075
	Sg vs. Fb	0.3333	0.1314 to 0.5353	**	0.0014
	In vs. Fb	0.21	0.008050 to 0.4119	*	0.0399

Tissue-specific gene expression patterns were analyzed by using Brown-Forsythe and Welch ANOVA followed by Tamhane's T2 multiple comparisons test. Mean transcript levels of *Nlug-desatB* and *Nlug-desatD* in different tissues of BPH were analyzed by using ordinary one-way ANOVA followed with Tukey's test.

Supplemental Table S6. Differences in specific fatty acid levels between a control group and each treatment group.

Brown and Welch ANOVA tests followed by Dunnett's T3 multiple comparisons test

Dunnett's T3 multiple comparisons test			**C12:0**		
Control vs. Treatment	Summary	Adjusted P Value	Mean Diff.	95.00% CI of diff.	Significant?
dsGFP vs. dsCytb5r	ns	0.7922	-0.05514	-0.1910 to 0.08069	No
dsGFP vs. dsdesatE	ns	0.9201	0.04387	-0.09196 to 0.1797	No
dsGFP vs. dsdesatA1-a	ns	0.9994	-0.01843	-0.1543 to 0.1174	No
dsGFP vs. dsdesatD	ns	0.7143	0.06063	-0.07521 to 0.1965	No
dsGFP vs. dsdesatB	ns	0.0623	0.1309	-0.004980 to 0.2667	No
dsGFP vs. dsIfc	ns	0.505	0.07479	-0.06105 to 0.2106	No
dsGFP vs. dsdesatA1-c	ns	0.0852	0.1237	-0.01217 to 0.2595	No
dsGFP vs. dsdesatC	ns	0.7238	0.05998	-0.07586 to 0.1958	No
dsGFP vs. dsdesatA1-b					
dsGFP vs. dsdesatA2	ns	0.0606	0.1315	-0.004323 to 0.2674	No
Dunnett's T3 multiple comparisons test			**C14:0**		
Control vs. Treatment	Summary	Adjusted P Value	Mean Diff.	95.00% CI of diff.	Significant?
dsGFP vs. dsCytb5r	ns	0.6496	-0.3649	-1.563 to 0.8332	No
dsGFP vs. dsdesatE	ns	0.9305	0.1514	-0.6210 to 0.9238	No
dsGFP vs. dsdesatA1-a	ns	0.9999	-0.02595	-1.090 to 1.038	No
dsGFP vs. dsdesatD	ns	0.85	0.1775	-0.5804 to 0.9353	No
dsGFP vs. dsdesatB	ns	0.1139	0.5659	-0.1936 to 1.325	No
dsGFP vs. dsIfc	*	0.0475	0.7841	0.01429 to 1.554	Yes
dsGFP vs. dsdesatA1-c	*	0.0378	0.8365	0.07577 to 1.597	Yes
dsGFP vs. dsdesatC	ns	0.2948	0.4677	-0.4162 to 1.352	No
dsGFP vs. dsdesatA1-b	*	0.0167	1.113	0.3463 to 1.879	Yes
dsGFP vs. dsdesatA2	ns	0.075	0.6871	-0.09316 to 1.467	No
Dunnett's T3 multiple comparisons test			**C16:0**		
Control vs. Treatment	Summary	Adjusted P Value	Mean Diff.	95.00% CI of diff.	Significant?
dsGFP vs. dsCytb5r	ns	0.6684	-15.28	-73.85 to 43.30	No
dsGFP vs. dsdesatE	ns	0.9994	2.68	-25.77 to 31.14	No
dsGFP vs. dsdesatA1-a	ns	0.9998	0.9501	-26.72 to 28.62	No
dsGFP vs. dsdesatD	ns	0.8662	6.198	-20.07 to 32.46	No
dsGFP vs. dsdesatB	ns	0.0992	19.59	-5.491 to 44.68	No
dsGFP vs. dsIfc	*	0.0406	26.8	1.825 to 51.78	Yes
dsGFP vs. dsdesatA1-c	*	0.028	29.97	5.132 to 54.81	Yes
dsGFP vs. dsdesatC	ns	0.1598	18.87	-8.845 to 46.59	No
dsGFP vs. dsdesatA1-b	*	0.0151	38.1	12.82 to 63.37	Yes
dsGFP vs. dsdesatA2	ns	0.0948	20.72	-4.724 to 46.17	No
Dunnett's T3 multiple comparisons test			**C16:1**		
Control vs. Treatment	Summary	Adjusted P Value	Mean Diff.	95.00% CI of diff.	Significant?
dsGFP vs. dsCytb5r	ns	0.8877	-0.2295	-1.303 to 0.8439	No
dsGFP vs. dsdesatE	ns	0.9995	0.07453	-0.8261 to 0.9751	No
dsGFP vs. dsdesatA1-a	ns	0.3509	0.3662	-0.5728 to 1.305	No
dsGFP vs. dsdesatD	ns	0.6314	0.2346	-0.7415 to 1.211	No
dsGFP vs. dsdesatB	ns	0.1904	0.5343	-0.5461 to 1.615	No
dsGFP vs. dsIfc	ns	0.1644	0.556	-0.4151 to 1.527	No
dsGFP vs. dsdesatA1-c	ns	0.1425	0.6096	-0.4066 to 1.626	No
dsGFP vs. dsdesatC	ns	0.2209	0.472	-0.4457 to 1.390	No
dsGFP vs. dsdesatA1-b					
dsGFP vs. dsdesatA2	ns	0.1678	0.5715	-0.4989 to 1.642	No

Dunnett's T3 multiple comparisons test			C18:0		
Control vs. Treatment	Summary	Adjusted P Value	Mean Diff.	95.00% CI of diff.	Significant?
dsGFP vs. dsCytb5r	ns	>0.9999	-0.04414	-3.894 to 3.806	No
dsGFP vs. dsdesatE	ns	0.6114	0.9451	-1.781 to 3.671	No
dsGFP vs. dsdesatA1-a	ns	0.2123	-2.476	-6.903 to 1.950	No
dsGFP vs. dsdesatD	ns	0.4005	1.254	-1.515 to 4.022	No
dsGFP vs. dsdesatB	ns	0.1053	1.787	-0.6065 to 4.181	No
dsGFP vs. dsIfc	*	0.0345	2.638	0.3191 to 4.957	Yes
dsGFP vs. dsdesatA1-c	*	0.0231	2.929	0.6030 to 5.255	Yes
dsGFP vs. dsdesatC	ns	0.0961	1.99	-0.4572 to 4.437	No
dsGFP vs. dsdesatA1-b	ns	0.9997	0.2955	-6.998 to 7.589	No
dsGFP vs. dsdesatA2	ns	>0.9999	0.03953	-2.910 to 2.989	No

Dunnett's T3 multiple comparisons test			C18:1		
Control vs. Treatment	Summary	Adjusted P Value	Mean Diff.	95.00% CI of diff.	Significant?
dsGFP vs. dsCytb5r	ns	0.7478	-13.08	-70.79 to 44.63	No
dsGFP vs. dsdesatE	ns	0.9996	2.317	-28.58 to 33.22	No
dsGFP vs. dsdesatA1-a	ns	0.9739	3.713	-19.83 to 27.26	No
dsGFP vs. dsdesatD	ns	0.8796	6.163	-20.92 to 33.24	No
dsGFP vs. dsdesatB	ns	0.0922	18.86	-4.732 to 42.45	No
dsGFP vs. dsIfc	*	0.0348	26.41	3.053 to 49.77	Yes
dsGFP vs. dsdesatA1-c	*	0.0298	27.42	4.224 to 50.62	Yes
dsGFP vs. dsdesatC	ns	0.075	20.45	-2.870 to 43.78	No
dsGFP vs. dsdesatA1-b	*	0.014	38.06	13.96 to 62.16	Yes
dsGFP vs. dsdesatA2	ns	0.1166	17.67	-5.725 to 41.07	No

Dunnett's T3 multiple comparisons test			C18:2		
Control vs. Treatment	Summary	Adjusted P Value	Mean Diff.	95.00% CI of diff.	Significant?
dsGFP vs. dsCytb5r	ns	0.5735	-5.35	-25.37 to 14.67	No
dsGFP vs. dsdesatE	ns	0.9996	-0.6449	-10.10 to 8.806	No
dsGFP vs. dsdesatA1-a	ns	0.9891	0.7422	-4.817 to 6.301	No
dsGFP vs. dsdesatD	ns	0.8882	1.301	-4.504 to 7.107	No
dsGFP vs. dsdesatB	ns	0.0537	6.37	-0.2141 to 12.95	No
dsGFP vs. dsIfc	ns	0.0718	4.916	-0.6131 to 10.45	No
dsGFP vs. dsdesatA1-c	*	0.0239	8.392	2.211 to 14.57	Yes
dsGFP vs. dsdesatC	ns	0.118	4.072	-1.502 to 9.645	No
dsGFP vs. dsdesatA1-b	*	0.0227	8.235	2.215 to 14.25	Yes
dsGFP vs. dsdesatA2	ns	0.333	2.905	-2.889 to 8.699	No

Differences in specific fatty acid levels between a control group (dsGFP-BPH) and each treatment group were determined by Brown-Forsythe and Welch ANOVA followed by Dunnett’s T3 multiple comparisons test. “ns”, no significance.

Supplemental Table S7. Primers used for cloning, qRT-PCR and T7 adapted primers used for downstream dsRNA synthesis steps.

Gene	Description	Forward primer (5'-…-3')	Reverse primer (5'-…-3')	Product sizes (bp)
Nlug-Cytb5r	Cloning	TGTCGTGCAACTAACAGGGA	CTGCTACACCATTCGTGGGA	1513
Nlug-desatE		TTGCATCCATCCATACGGCA	GTCCAAGAAACCGGGCCTTA	1709
Nlug-desatA1-a		TGTTTAGTCCGAGGACAAGGAA	ATGCAAGTAGACAGGTGGGC	1673
Nlug-desatD		GCCTGGTGTGTCACAAGGAT	AGCATTCAGTGTACGACACTTTG	1056
Nlug-desatB		CAGAATGGGCGCTCAGCTA	GTGACAACCGTTCACCGTCA	1000
Nlug-ifc		TGCAGTATTGTGTTACCGGCT	TGCACTTGGCTGGCAAAAAT	1225
Nlug-desatA2-a		TGAAGTGCCCGCCTTAGGTA	CGCCTCAGATGTTGAAGGGT	1237
Nlug-desatC		AGTGCTAAGTCGAGTGACAGC	TGTTGCATTGTAGCTTCGACA	1175
Nlug-desatA1-b		GCAACCCCTCGTTCTCAGTT	AGCAAAATGAGTTGCGCGAT	1342
Nlug-desatA2-b		CCAATCGCAAAAGACGGCAT	CGATCAATTTCTTGTCGCCCG	1280
Nlug-Cytb5r	qRT-PCR	CAGCTCACCATCATCCAGAC	ACCACGGGAATGTCTTCTTC	123
Nlug-desatE		GAGCATATTGCTGCCGATAA	AATGGTGGCTGAGTTGATGA	133
Nlug-desatA1-a		CTTCACCACCAGATTCATCG	AGATCTTGATCGTCCCATCC	150
Nlug-desatD		TGCACAAACAACAATCACCA	CACCGCCATGTACGAATAAT	112
Nlug-desatB		GCCTACGACCTCAAGAGTCC	AGGTGATCGTCCCAGGTAAG	93
Nlug-ifc		TATTATGGCTGCCTCAATGC	CTGAAGCAATTTCACGAACC	112
Nlug-desatA2-a		AAATACCAGGAGACCGATGC	CGGAGATGTCGATACCCTTT	127
Nlug-desatC		CGACCTCAAAGAAGCCAAGCC	TAGGTGCGTCCTTCGATCCC	131
Nlug-desatA1-b		ACATGTGCTCAGGATTTGGA	GGGAAATTTGCCTTGTAGGA	76
Nlug-desatA2-b		CTGGAGCAGAAGTCATCCCT	CGCTGTTGTTGTTGTTGTTG	103
RPS11		CCGATCGTGTGGCGTTGAAGGG	ATGGCCGACATTCTTCCAGGTCC	159
RPS15		TAAAAATGGCAGACGAAGAGCCCAA	TTCCACGGTTGAAACGTCTGCG	150

Gene	Description	Forward primer (5'-…-3')	Reverse primer (5'-…-3')	Product sizes (bp)
Nlug-Cytb5r	dsRNA synthesis	GGATCCTAATACGACTCACTATAGG ACGAGTCGTCACACCTGAAC	GGATCCTAATACGACTCACTATAGG ATGAAACCGGCCGTGTAGTT	315
Nlug-desatE		GGATCCTAATACGACTCACTATAGG ACTTGTTTGTCAGGGATCGG	GGATCCTAATACGACTCACTATAGG CGGCAGCAATATGCTCACTA	312
Nlug-desatA1-a		GGATCCTAATACGACTCACTATAGG GGTTAATGATCCGCAAGCAT	GGATCCTAATACGACTCACTATAGG GCTGCACTGTTTACAAGCCA	303
Nlug-desatD		GGATCCTAATACGACTCACTATAGG GCTCATCGTCTATGGTCGCA	GGATCCTAATACGACTCACTATAGG GGACATGTCGATCGCTTTGC	305
Nlug-desatB		GGATCCTAATACGACTCACTATAGG CAAATTGCCACTGCAGCTAA	GGATCCTAATACGACTCACTATAGG CCGGGAATATGAAGCAAAAA	352
Nlug-ifc		GGATCCTAATACGACTCACTATAGG TATTCATCCTGTGGCTGGGC	GGATCCTAATACGACTCACTATAGG TCTCTTGATGCGGGCGTATG	330
Nlug-desatA2-a		GGATCCTAATACGACTCACTATAGG GACAGCTGTTCGAGGAGGAC	GGATCCTAATACGACTCACTATAGG AGTCTGTGAACTCCCGCTGT	282
Nlug-desatC		GGATCCTAATACGACTCACTATAGG AGGTTGGCACAACTTCCATC	GGATCCTAATACGACTCACTATAGG CATTCTGCACCTTTCTGCAA	269
Nlug-desatA1-b		GGATCCTAATACGACTCACTATAGG CAAGAAGCCCGAACAGAAAC	GGATCCTAATACGACTCACTATAGG TGCACTCTGTGATCTCTGGC	356
Nlug-desatA2-b		GGATCCTAATACGACTCACTATAGG GTGGAGAAATGTTGCTGCCT	GGATCCTAATACGACTCACTATAGG CGGTGTCTGTGAACTTGTGG	337
GFP		GGATCCTAATACGACTCACTATAGG AAGGGCGAGGAGCTGTTCACCG	GGATCCTAATACGACTCACTATAGG CAGCAGGACCATGTGATCGCGC	707
